# Editorial: The Role of Astroglia and Oligodendroglia in CNS Development, Plasticity, and Disease – Novel Tools and Investigative Approaches

**DOI:** 10.3389/fncel.2022.901820

**Published:** 2022-05-09

**Authors:** Enrica Boda, Francesca Boscia, Christian Lohr

**Affiliations:** ^1^Department of Neuroscience Rita Levi-Montalcini, Neuroscience Institute Cavalieri Ottolenghi (NICO), University of Turin, Turin, Italy; ^2^Division of Pharmacology, Department of Neuroscience, Reproductive Sciences and Dentistry, School of Medicine, Federico II University of Naples, Naples, Italy; ^3^Division of Neurophysiology, University of Hamburg, Hamburg, Germany

**Keywords:** astrocytes, oligodendrocytes, methods, experimental models, CNS development, CNS plasticity, CNS disease

The development of novel tools and methodologies to probe and manipulate glial cell functions and signaling has recently allowed an unprecedented level of comprehension of astro- and oligodendroglia contribution to CNS development, homeostasis, and plasticity. Both cell types critically contribute to neuronal function throughout the lifespan by providing metabolic support, promoting synchronization of neuronal networks and being involved in the homeostasis of ions, neurotransmitters, reactive oxygen species, and metabolites. Astroglia support synaptogenesis and synapse plasticity, whereas oligodendroglia assure developmental myelination, sustain axon maturation and integrity, and plastically respond to experience with *de-novo* myelin deposition or remodeling (Verkhratsky and Nedergaard, 2018; Kuhn et al., 2019). Studies have also provided evidence of a remarkable level of functional, molecular and developmental heterogeneity (Marques et al., 2016; Cerrato et al., 2018; Khakh and Deneen, 2019; Batiuk et al., 2020; Sherafat et al., 2021; Seeker and Williams, 2022) and unexpected interactions with immune cells (including microglia, peripheral macrophages, and T cells) for both astrocytes and oligodendroglia (Ponath et al., 2018; Kirby and Castelo-Branco, 2021). This latter aspect contributes to astro-/oligodendroglia response to injury. In this respect, recent technological advances also allowed to understand that dysregulations affecting the crosstalk between astro-/oligodendroglia and other cellular partners, including neurons, can be primarily involved in the initiation and progression of neurodevelopmental, psychiatric and neurodegenerative disorders, revealing their potential as therapeutic targets (Salmaso et al., 2014; Boda, 2021; Boscia et al., 2021; Healy et al., 2022; Lee et al., 2022).

This Research Topic Issue consists of 16 articles, i.e., seven literature reviews/minireviews, 8 original research articles and one “Technology and Code” manuscript, presenting or exploiting state-of-the-art methodological approaches as well as novel tools and experimental models to understand the diverse roles of astrocytes and oligodendroglia in CNS development, function and dysfunction.

Specifically, as regards novel methods to study astrocyte physiology and morphology, in their research article, Köhler et al. provide a new approach to study ATP metabolism in brain cells using a combination of two ATP nanosensors with different affinities that allow for the measurement of basal cytosolic concentration at rest as well as changes following challenges. In Minge et al., high resolution microscopy is employed to study the morphology of fine astrocyte processes by analyzing the tissue volume fraction occupied by astrocytes and the astrocytic process density. Schweigmann et al. design new liquid crystal polymer surface electrodes for recording and stimulating neuronal activity with a central window that allows for simultaneous imaging. They combine the surface electrodes with two-photon Ca^2+^ imaging in *in vivo* experiments, in which they stimulate neurons and record Ca^2+^ signals in astrocytes.

Three reviews summarize methodological aspects to study astrocyte properties. Stephan et al. provide an overview of state-of-the-art techniques to record, visualize and analyze gap junctions coupling astrocytes or established between astrocytes and other types of glial cells, so-called panglial coupling. Ca^2+^ signaling is a key feature of astrocytes and the development of highly sensitive genetically encoded Ca^2+^ indicators has boosted physiological research on astrocytes. While the review by Lia et al. focusses on recent advances regarding mechanisms and functions of Ca^2+^ signaling, in particular in astrocytic microdomains, Lohr et al. present a brief compendium of genetically encoded Ca^2+^ indicators used in glial cell research that compares the most important properties of the indicators.

By generating novel mouse mutants, Romeo et al. show that the astrocyte-specific deletion of the low-density lipoprotein receptor-related protein 1 (LRP1), a transmembrane receptor with multiple possible ligands, causes a transient delay in hippocampal astrocyte maturation at an early developmental timepoint, which in turn results in a reduced activity of neurons within the hippocampus. Moreover, by exploiting an *in vitro* model of the tripartite synapses, the same group, Romeo et al. show that the astrocyte-specific deletion of LRP1 negatively influences neuronal network activity and synaptogenesis, and propose that this effect may be mediated by an alteration of the expression of the glutamate transporter and of the release of cytokines in LRP1-deleted astrocytes.

Finally, in an in silico study, Fritschi et al. address the question of how astrocytes and neurons are involved in schizophrenia.

The “oligodendrocyte” section includes three reviews summarizing current and cutting-edge approaches. Heflin and Sun review state-of-art approaches for studying neuron-oligodendrocyte interactions and activity-dependent myelination with both temporal and spatial precision. They specifically highlight novel transgenic mouse models and techniques such as electrophysiology, *in vivo* imaging, and 2-photon uncaging. Marangon et al. provide an extensive overview of the advantages and limitations of the current and emerging techniques to study (re)myelination *in vitro*. Beside the classical (re)myelination assays with rodent oligodendrocyte precursor cell (OPC)-neuron co-cultures and *ex vivo* organotypic cultures, the authors discuss the most recent approaches based on the use of human iPSC-derived OPCs and 3D organoids. The implications of the use of personalized humanized models and their optimization for the identification of remyelinating agents are also discussed. Galichet et al. provide a collection of recently used approaches including functional genetics, transcriptomics, imaging, electrophysiology and optogenetics, to study OPC biology, as well as their morphological, functional and molecular heterogeneity. They also discuss the multitude of culture systems used to generate and maintain OPCs *in vitro*, including 2D and 3D techniques, using primary animal or human cell lines or OPCs derived from induced pluripotent stem cells.

In a “Technology and Code” manuscript, Xu et al. describe a novel software tool (Oligo-Track) for an automated image analysis and *in vivo* tracking of oligodendrocytes over time in the mammalian brain with multi-photon microscopy. The method described has significant advantages in speeding up the analysis of oligodendrocyte number, distribution, and size over other approaches and will be available to the community.

Reiche et al. exploit a combination of data mining/bioinformatic analyses, histological analyses and *in vitro* assays to investigate the role of C21ORF91, found overexpressed in Down Syndrome (DS) iPSCs and considered a key modulator of aberrant CNS development in DS, in oligodendroglia. By reproducing C21orf91 overexpression in cultured primary OPCs, the authors show that C21orf91 critically regulates OPC myelination capacity. Further, C21orf91 overexpression results in the appearance of cells endowed with both oligodendroglia and astroglia features, rising the hypothesis that a developmental fate switch could contribute to white matter alterations in DS.

In their research article, Hardt et al. investigate the expression changes of auxiliary transmembrane AMPA receptor related proteins (TARPs) during postnatal maturation and the functional consequences on AMPA receptor properties and Ca^+2^ permeability in NG2 glia. The increased proportion of Ca^2+^ permeable AMPA receptors at the soma during postnatal maturation is accompanied by the downregulation of TARP γ8, suggesting that AMPA receptors Ca^2+^ permeability during postnatal maturation may be affected by the interaction with TARPs.

Finally, Tiberi and Chiurchiù review the role of a new class of intercellular mediators, i.e., the specialized pro-resolving lipid mediators (SPMs), formerly studied in the context of the resolution of peripheral inflammation, in the regulation of astrocytes/oligodendroglia state and cross-talk with microglia and neurons, highlighting their relevance and therapeutic potential in the context of neuroinflammatory and neurodegenerative diseases.

In summary, the manuscripts gathered in this Research Topic provide an updated insight into *in silico*/bioinformatic, *in vitro, ex vivo*, and *in vivo* approaches that have been recently developed to study the functions of astro-/oligodendroglia, and highlight the potency of these cutting-edge approaches to address novel questions about the role of these cells in CNS development, function and disease. We hope that this Research Topic will stimulate discussion and new research directions to move forward on glial cell studies with relevant implications for the development of glia-focused therapeutic approaches for CNS disease. As Guest Editors, we thank all the colleagues who accepted the invitation to participate in this Research Topic and share their manifold expertise and insights and to the Editorial Staff of Frontiers in Cellular Neuroscience—Section Non-Neuronal Cells for their assistance.

## Author Contributions

EB, FB, and CL contributed to the manuscript conceptualization, writing, review and editing, and to funding acquisition. All authors contributed to the article and approved the submitted version.

## Funding

This work was supported by the Fondazione Italiana Sclerosi Multipla (FISM) 2019/PR-Multi/003 grant, the Cassa di Risparmio di Torino (CRT) Foundation grant (ID 2021.0657), and local funds of University of Turin, to EB; Fondazione Italiana Sclerosi Multipla FISM 2015/R/6 and Italian Ministry for University and Research PRIN 2017, 20175SA5JJ to FB. This study was also supported by the Italian Ministero dell'Istruzione, dell'Università e della Ricerca -MIUR project Dipartimenti di Eccellenza 2018–2022 to Department of Neuroscience Rita Levi Montalcini and by the Deutsche Forschungsgemeinschaft (SFB 1328, project number 335447717).

## Conflict of Interest

The authors declare that the research was conducted in the absence of any commercial or financial relationships that could be construed as a potential conflict of interest.

## Publisher's Note

All claims expressed in this article are solely those of the authors and do not necessarily represent those of their affiliated organizations, or those of the publisher, the editors and the reviewers. Any product that may be evaluated in this article, or claim that may be made by its manufacturer, is not guaranteed or endorsed by the publisher.
